# The Viral and Cellular MicroRNA Targetome in Lymphoblastoid Cell Lines

**DOI:** 10.1371/journal.ppat.1002484

**Published:** 2012-01-26

**Authors:** Rebecca L. Skalsky, David L. Corcoran, Eva Gottwein, Christopher L. Frank, Dong Kang, Markus Hafner, Jeffrey D. Nusbaum, Regina Feederle, Henri-Jacques Delecluse, Micah A. Luftig, Thomas Tuschl, Uwe Ohler, Bryan R. Cullen

**Affiliations:** 1 Department of Molecular Genetics and Microbiology, Duke University, Durham, North Carolina, United States of America; 2 Duke Institute for Genome Sciences and Policy, Duke University, Durham, North Carolina, United States of America; 3 Department of Microbiology-Immunology, Northwestern University, Feinberg School of Medicine, Chicago, Illinois, United States of America; 4 Laboratory of RNA Molecular Biology, The Rockefeller University, New York, New York, United States of America; 5 German Cancer Research Center, Department of Virus-Associated Tumours, Heidelberg, Germany; 6 Department of Biostatistics and Bioinformatics, Duke University, Durham, North Carolina, United States of America; Oregon Health and Science University, United States of America

## Abstract

Epstein-Barr virus (EBV) is a ubiquitous human herpesvirus linked to a number of B cell cancers and lymphoproliferative disorders. During latent infection, EBV expresses 25 viral pre-microRNAs (miRNAs) and induces the expression of specific host miRNAs, such as miR-155 and miR-21, which potentially play a role in viral oncogenesis. To date, only a limited number of EBV miRNA targets have been identified; thus, the role of EBV miRNAs in viral pathogenesis and/or lymphomagenesis is not well defined. Here, we used photoactivatable ribonucleoside-enhanced crosslinking and immunoprecipitation (PAR-CLIP) combined with deep sequencing and computational analysis to comprehensively examine the viral and cellular miRNA targetome in EBV strain B95-8-infected lymphoblastoid cell lines (LCLs). We identified 7,827 miRNA-interaction sites in 3,492 cellular 3′UTRs. 531 of these sites contained seed matches to viral miRNAs. 24 PAR-CLIP-identified miRNA:3′UTR interactions were confirmed by reporter assays. Our results reveal that EBV miRNAs predominantly target cellular transcripts during latent infection, thereby manipulating the host environment. Furthermore, targets of EBV miRNAs are involved in multiple cellular processes that are directly relevant to viral infection, including innate immunity, cell survival, and cell proliferation. Finally, we present evidence that myc-regulated host miRNAs from the miR-17/92 cluster can regulate latent viral gene expression. This comprehensive survey of the miRNA targetome in EBV-infected B cells represents a key step towards defining the functions of EBV-encoded miRNAs, and potentially, identifying novel therapeutic targets for EBV-associated malignancies.

## Author Summary

Over 90% of adults worldwide are infected with Epstein-Barr virus (EBV). While EBV infection is normally controlled by a healthy immune system, in immuno-compromised individuals, EBV can cause serious disease and/or cancer. During infection, EBV expresses viral microRNAs (miRNAs) and induces the expression of specific cellular miRNAs. In general, miRNAs inhibit target gene expression by binding to complementary regions on target messenger RNAs (mRNA). While cellular miRNAs regulate important biological processes such as cell growth and differentiation, and many miRNAs have been linked to cancer progression, the functions of EBV miRNAs are largely unknown. To identify targets of EBV miRNAs and cellular miRNAs in EBV-infected cells, we used a high-throughput method based on next-generation sequencing technology to give a global picture of miRNA-regulated gene expression. Our analysis showed that over 500 mRNAs can be regulated by viral miRNAs, many of which are directly relevant to EBV infection. This study provides a comprehensive survey of viral and cellular miRNA targets in B cells, which is a positive step towards identifying novel therapeutic targets for EBV-associated cancers.

## Introduction

Epstein-Barr virus (EBV) is a ubiquitous human γ-herpesvirus that can induce the proliferation of resting B lymphocytes *in vivo*. Following primary infection, EBV establishes a lifelong latent infection and, in the absence of effective immune surveillance, can induce lymphoproliferative diseases. EBV is associated with numerous B cell malignancies including Hodgkin's lymphoma (HL), Burkitt's lymphoma (BL), and diffuse large B cell lymphoma (DLBCL) as well as post-transplant lymphoproliferative disorders (PTLD) [1]. *In vitro*, EBV immortalizes primary human B lymphocytes, establishing a type III latency program characterized by the expression of a subset of viral gene products including six EBV nuclear antigens (EBNAs), three latent membrane proteins (LMPs), and non-coding RNAs including EBV-encoded small RNAs (EBERs) and viral microRNAs (miRNAs) [1]–[6]. The resulting lymphoblastoid cell lines (LCLs) serve as an *in vitro* model for EBV-associated lymphoproliferative diseases and share many of the characteristics of PTLD in terms of latent viral gene expression [1].

miRNAs are ∼22 nucleotide (nt) non-coding RNAs that post-transcriptionally regulate gene expression. miRNAs are expressed by all metazoans as well as a number of viruses. EBV encodes 25 miRNA precursors (three BHRF1 pre-miRNAs and 22 BART pre-miRNAs), which are located in two regions of the genome [2]–[6]. Expression of the BHRF1 miRNAs is restricted to type III latency, which is observed in LCLs and PTLD, while BART miRNAs are variably expressed in all latency stages [3], [7], [8]. EBV miRNA biogenesis follows the canonical cellular miRNA biogenesis pathway, initiating in the nucleus with primary miRNA stem-loops that are cleaved by Drosha, exported into the cytoplasm, and cleaved by Dicer into ∼22 nt RNA duplexes (reviewed in [9]). One strand of the duplex is incorporated into the RNA-induced silencing complex (RISC), which minimally consists of a mature miRNA and an Argonaute (Ago) protein. The mature miRNA guides RISC to complementary sites predominantly in 3′UTRs of target mRNAs, resulting in translational repression and/or mRNA degradation (reviewed in [10]). Especially important for miRNA targeting are nucleotides (nt) 2-8, minimally nt 2-7, of the mature miRNA, termed the “seed” sequence, which generally binds with perfect Watson-Crick base pairing to target mRNAs [10], [11].

In addition to expressing viral miRNAs, EBV infection induces the expression of several cellular miRNAs, including miR-155, miR-146a, and miR-21 [12]–[15]. Recent studies suggest that miR-146a functions as a tumor suppressor since genetic ablation of miR-146a in mice induces myeloid tumors [16]. In contrast, both miR-155 and miR-21 are over-expressed in a number of cancers, including B cell lymphomas, and when over-expressed in transgenic mouse models, these miRNAs induce B cell tumors [17]–[19]. Recently, miR-155 has been shown to be required for the growth and survival of LCLs *in vitro* [20]. In fact, miR-155 regulated pathways are likely of importance to oncogenic herpesvirus biology in general since the related γ-herpesvirus Kaposi's sarcoma-associated herpesvirus (KSHV), which is linked to a number of B cell malignancies, as well as Marek's disease virus (MDV), which causes T cell lymphomas in chickens, both encode functional analogs of miR-155 [21]–[24].

We and others have hypothesized that viral miRNAs as well as cellular miRNAs induced by viral infection have a direct impact on the cellular gene expression pattern that favors the establishment and/or maintenance of latent infection [9], [25], [26]. Identifying the targets of viral and cellular miRNAs is a key step in elucidating their functional roles during infection as well as their potential contributions to viral pathogenesis and lymphomagenesis. Several studies point to important functional roles for EBV miRNAs during the viral life cycle, including immune evasion, cell survival and proliferation, and control of the latent/lytic switch. miR-BART2-5p, for example, targets the lytic viral DNA polymerase encoded by BALF5 [4], [27]. miR-BART2-5p is also reported to downregulate expression of MICB, a natural killer (NK) cell ligand, which contributes to immune evasion [28]. Similarly, miR-BHRF1-3 downregulates CXCL11, an interferon-inducible chemokine and T cell attractant [29]. Finally, LMP1 and LMP2A, which not only contribute to the survival, proliferation and transformation of EBV-infected cells, but are also immunogenic, are both reported targets of EBV BART miRNAs [30], [31].

Recent studies performed with EBV miRNA deletion mutants indicate that EBV miRNAs contribute to, but are not essential for, LCL formation *in vitro* [32]–[34]. The EBV B95-8 laboratory strain bears a deletion within the BART region and encodes only five of the 22 BART miRNAs. This virus can still immortalize B cells *in vitro*, and inactivation of the remaining BART miRNAs has little effect on LCL outgrowth [34]. However, mutational inactivation of the BHRF1 miRNAs inhibits LCL outgrowth, reduces the ability of LCLs to progress from G1 to S phase during the cell cycle, and affects EBV latent gene expression [32]–[34]. The viral miRNA targets responsible for these phenotypes are not yet defined. Indeed, since the identification of the first EBV miRNAs in 2004, only ten targets of EBV miRNAs have been experimentally validated [4], [27]–[31], [35]–[38]. To date, the largest screen of EBV miRNA targets identified 44 candidate mRNA targets in B cells, only two of which were further validated [35]. As cellular miRNAs are predicted to individually target more than 100 mRNAs [39], and EBV expresses at least 25 miRNAs, these 44 candidate targets represent only ∼1% of the potential viral miRNA targetome.

Here, we report the use of photoactivatable-ribonucleoside enhance crosslinking and immunoprecipitation (PAR-CLIP) to interrogate the viral and cellular miRNA targetome in latently infected EBV-B95-8 LCLs. We identified over 500 canonical, 5′ seed target sites for EBV-B95-8 miRNAs and over 3,000 seed target sites for cellular miRNAs in these cells. Binding sites for cellular miRNAs in EBV transcripts were also detected, and we uncovered several non-canonical target sites, providing new insight into the dynamics of miRNA regulation. We show that EBV-B95-8 miRNAs predominantly target cellular transcripts during latent infection and experimentally validate a number of mRNA targets for EBV miRNAs with roles in innate immunity, stress response, and cell signaling.

## Results

### miRNAs expressed in LCLs

To identify miRNA targets in LCLs, it was important to first define the LCL miRNA transcriptome at the sequence level. Therefore, we profiled all small RNAs, cellular and viral, expressed in EBV-B95-8-infected LCLs using the Illumina sequencing platform. We used the EBV-B95-8 strain primarily due to the availability of miRNA deletion mutants that were generated in the B95-8 background (see below). High-throughput sequencing of four LCLs (SDLCL, LCL35, EF3D-AGO2 expressing a FLAG-tagged Ago2, and LCL-BAC) yielded over 28 million reads, of which 89.3% aligned to the human genome and 6.9% aligned to the EBV B95-8 genome (Table S1). Reads were then mapped to known human and viral pre-miRNAs present in miRBASE v16.0. We identified miRNAs from 438 human pre-miRNAs and eight viral pre-miRNAs that were expressed in the four LCLs (Tables S2-S5).

**Figure 1:**
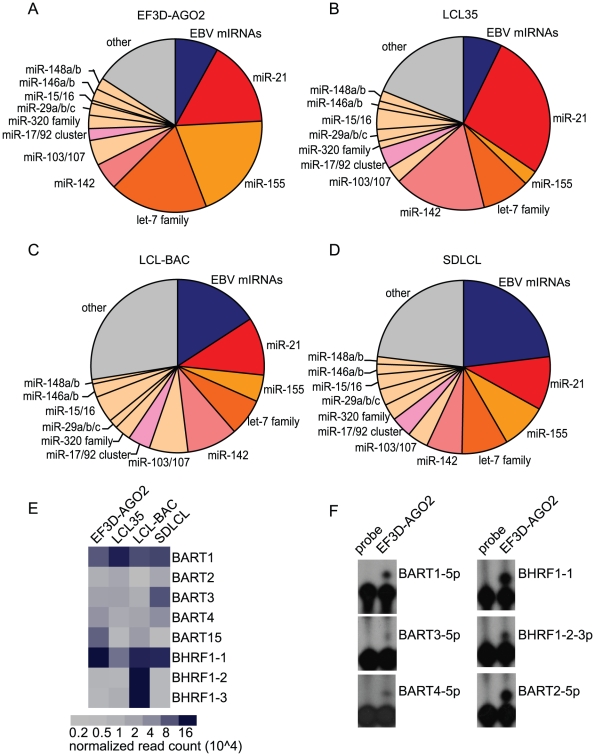
Cellular and viral miRNAs detected in LCLs by deep sequencing. A–D. miRNAs present in four LCLs (EF3D-AGO2, LCL35, LCL-BAC, SDLCL) were detected by deep sequencing. Shown is the distribution of reads mapping to cellular (orange, grey) and viral (dark blue) pre-miRNAs; the most abundant cellular miRNAs are highlighted. miR-17/92 includes reads mapping to miR-17, 18a, 19, 20a, and 92a. E. EBV miRNA expression as determined by deep sequencing. Read counts were normalized to the total number of reads mapping to pre-miRNAs in each library. F. Primer extension analysis detects six EBV miRNAs expressed in EF3D-AGO2.

The most abundant cellular miRNAs expressed in LCLs included miR-21, let-7(a-i), miR-142, miR-155, miR-103, miR-320a/b/c/d, and miR-146a/b (Figure 1A–D). Together, these miRNAs accounted for ∼50% of all miRNAs detected. Notably, high expression for many of these cellular miRNAs is congruent with other miRNA profiling studies done with EBV-infected B cells [13], [40], [41]. For example, elevated levels of miR-21, miR-155, miR-146a, and miR-146b have been reported in EBV+ LCLs and BL cells in type III latency and induction of miR-146a via NFκB activation has been linked to LMP1 expression [13], [14]. Moreover, EBV infection strongly stimulates miR-155 expression, which is critical for the growth of LCLs *in vitro* [13]–[15], [20], [41]-[43].

EBV-encoded miRNAs accounted for 7-22% of the total miRNAs expressed in LCLs (Figure 1A–D). Ten mature miRNAs originating from all eight EBV pre-miRNAs encoded by the B95-8 strain were identified (Table 1; Fig. 1E). We also detected low levels of miRNA passenger strands, including miR-BART15-5p, miR-BART2-3p, and miR-BART4-3p (Tables S2-S5). Consistent with the latency III gene expression pattern in LCLs, we detected all BHRF1 miRNAs, which arise from Cp/Wp-initiated latent transcripts [3], [8]. Although BART miRNAs are predominantly expressed at high levels during latency II, such as in EBV-associated nasopharyngeal carcinomas (NPC) and NPC-derived cells [3], [5], [6], we also detected miRNAs from all five EBV-B95-8 BART pre-miRNAs, congruent with previous reports indicating that the BART miRNAs are expressed in all stages of EBV latency [7], [8]. Based on read counts, miR-BHRF1-1 and miR-BART1-5p were expressed at higher levels than other EBV miRNAs (Tables S2-S5, Figure 1E). We confirmed the expression of six different EBV miRNAs by primer extension analysis (Figure 1F).

**Table 1:** Predominant isoforms of EBV miRNAs as detected by deep sequencing.

miRNA	Sequence
ebv-mir-bart1-5p	TCTTAGTGGAAGTGACGTGCTGTG(A)
ebv-mir-bart1-3p	TAGCACCGCTATCCACTATGTC
ebv-mir-bart15-5p (*)	AGGGAAACATGACCACCTGAAGTCT
ebv-mir-bart15-3p	GTCAGTGGTTTTGTTTCCTTGA
ebv-mir-bart2-5p	TATTTTCTGCATTCGCCCTTGC
ebv-mir-bart2-3p (*)	AAGGAGCGATTTGGAGAAAATAAA
ebv-mir-bart3-5p (*)	ACCTAGTGTTAGTGTTGTGCTG
ebv-mir-bart3-3p	CGCACCACTAGTCACCAGGTGT
ebv-mir-bart4-5p	GACCTGATGCTGCTGGTGTGC(T)
ebv-mir-bart4-3p (*)	CACATCACGTAGGCACCAGGTGT
ebv-mir-bhrf1-1	TAACCTGATCAGCCCCGGAGTT(G)
ebv-mir-bhrf1-2-5p	AAATTCTGTTGCAGCAGATAGC
ebv-mir-bhrf1-2-3p	TATCTTTTGCGGCAGAAATTGA
ebv-mir-bhrf1-3	TAACGGGAAGTGTGTAAGCACA

(*) indicates the miRNA was previously annotated as the miRNA* or passenger strand and by deep sequencing here, accounts for less than 5% of the total reads mapping to the pre-miRNA. ebv-mir-bart1-3p and ebv-mir-bhrf1-2-3p were previously annotated in miRBase as miRNA* but do not meet the criteria.

Minor sequence variations occurred at the 3′ ends of both the viral and cellular miRNAs, which is consistent with other reports on miRNA deep sequencing of γ-herpesvirus-infected cells [44]–[47]. The 5′ ends were precise for all detected viral miRNAs (Table 1) as well as for the majority of cellular miRNAs; however, 5′ nt variations were observed for a few cellular miRNAs (i.e. miR-140-3p, miR-142-3p, miR-10a) (Tables S2–S5). Primary transcripts for miR-BHRF1-1 and other EBV miRNAs were previously reported to undergo adenosine (A) to inosine (I) editing [36]; indeed, one recent study estimated that 20% of human pri-miRNAs may be subjected to A to I editing by ADARs and can directly influence miRNA targeting [48]. Therefore, we also examined all reads mapping to EBV and cellular pre-miRNAs for A>G substitutions, but found no evidence of A to I editing in any mature viral miRNA sequences (data not shown).

### Identification of Ago2 binding sites using PAR-CLIP

Having established the EBV-B95-8 LCL miRNA transcriptome, we next sought to determine the targets for all viral and cellular miRNAs expressed in LCLs during latent EBV infection. To capture miRNA:mRNA target interactions in a comprehensive and high-throughput manner, we implemented the PAR-CLIP method combined with computational analysis to directly identify RISC-bound mRNAs [49], [50]. Using this method, we analyzed three of the four “wild-type” EBV-B95-8 LCLs from Figure 1 (EF3D-AGO2 (stably expressing a FLAG-tagged version of Ago2), LCL35, and LCL-BAC) and two additional LCLs, LCL-BAC-D1 and LCL-BAC-D3, infected with miRNA-knockout viruses lacking miR-BHRF1-1 or miR-BHRF1-3, respectively (Table S1). These miRNA-knockout viruses, and the phenotypes associated with mutational inactivation of the individual BHRF1 miRNAs, have been previously described [32].

LCLs were cultured in the presence of 4-thiouridine (4SU), a photoactivatable ribonucleoside analog which is readily taken up by cells and incorporated into nascent RNAs. The cells were then UV irradiated at 365 nm to cross-link RISC-bound RNAs. Cross-linked RNAs were immunopurified using either anti-FLAG (EF3D-AGO2) or anti-Ago2 antibodies (remaining four LCLs), digested with RNAse T1, and reverse transcribed to cDNA for deep sequencing. The incorporation of 4SU into nascent RNAs is a key component of PAR-CLIP. During reverse transcription, 4SU favors pairing with guanine (G) and consequently, is converted to a cytosine (C) during PCR amplification. Thus, in the final sequencing reads, a thymine to cytosine (T>C) conversion marks the cross-linked site, and generally, these T>C conversions occur 3′ or 5′ to a miRNA seed match site [49], [50].

Over 46 million reads were obtained from the five LCL PAR-CLIP libraries, and 10.3 million reads aligned at one unique location to either the human or EBV genome (Table S1). In order to extract as much information as possible from our sequencing data, we allowed for up to three mismatches including T>C conversions in our alignments. To identify miRNA-targeted regions, we used the PARalyzer toolkit, described in detail elsewhere [49]. Briefly, reads were grouped according to genomic location and read clusters with T>C signals above background were analyzed for canonical miRNA seed match sites. Such clusters represent miRNA:mRNA interaction sites, and unless otherwise noted, these sites minimally include: 7mer (nt 2-8) and 7mer1A (nt 2-7 with an A across from position one of the mature miRNA) seed matches [10], [11]. Compared to previous clustering strategies [50], our analysis of PAR-CLIP reads leads to a better resolution of miRNA binding sites, particularly those in close proximity to each other. For example, in EF3D-AGO2, the BACH1 3′UTR has at least 22 mapped clusters (Table S10), four of which are separated by ≤ 12 nt and, using our approach, could be assigned to different targeting miRNAs (Table S10).

### Clusters are 3′UTR biased and preferentially distributed towards 3′UTR termini

A total of 153,007 clusters were identified in all five PAR-CLIP libraries (Table S1), and 40.3% of these clusters were detected in at least two of the five libraries (Figure S1). We were encouraged by this high level of cluster overlap given the fact that the five LCLs were (i) generated from different donors, (ii) in culture for different lengths of time, (iii) infected with EBV B95-8, EBV B95-8-derived Bacmid, or BHRF1 miRNA mutant viruses, and (iv) the Ago2 immunoprecipitations were performed with different antibodies (i.e. anti-FLAG antibody vs antibody to endogenous Ago2). Interestingly, we observed greater cluster overlap for LCL-BAC and LCL-BAC-D1 compared to the other three libraries (Figure S1A). Although both LCL-BAC and LCL-BAC-D1 had fewer aligned reads and, therefore, fewer clusters than the other libraries (Table S1), a greater percentage of their clusters were detectable in other libraries. This suggests that the partial overlap observed for individual libraries may be due to non-saturating sequencing depth (despite the fact that we obtained a minimum of five million reads per sample using the then available deep sequencing technology).

To further investigate clusters that were identified in multiple libraries, we plotted the average read count for each cluster against the number of libraries containing that cluster (Figure S1C). Clusters with higher read counts were captured more consistently in multiple PAR-CLIP libraries than clusters with lower read counts. Interestingly, we observed a similar correlation when we plotted clusters that could be assigned to a targeting miRNA (see below) against miRNA expression level (Figure S1D). Clusters with seed matches to highly expressed miRNAs were captured more consistently in multiple PAR-CLIP libraries than clusters with seed matches to weakly expressed miRNAs. Together, these data suggest that clusters present in only a single library are more likely to represent interactions between low expressed miRNAs and mRNAs, weak miRNA:mRNA interactions, or possibly interactions between miRNAs and low expressed mRNAs, although we cannot rule out transient interactions. Furthermore, clusters present in multiple libraries are more likely to represent miRNA:mRNA interactions between highly expressed miRNAs and strong target sites on mRNAs. Based on these results, we focused our analysis on clusters that were present in at least two of the five libraries.

**Figure 2:**
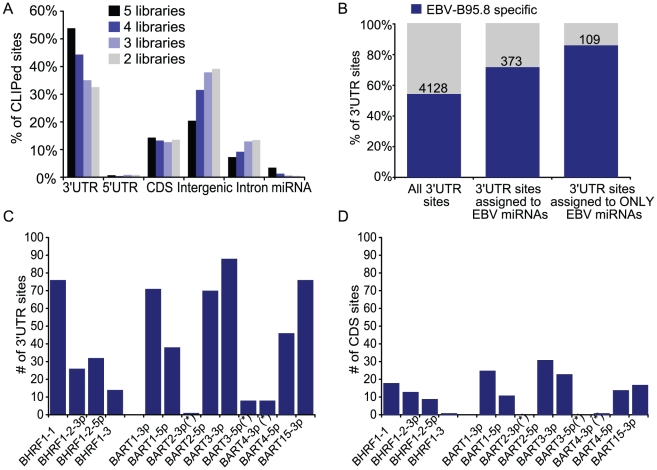
Distribution of PAR-CLIP Clusters reveals 3′UTR bias. A. Overlap of clusters in the five PAR-CLIP libraries. Shown is the breakdown of clusters mapping to 3′UTRs, 5′UTRs, coding regions (CDS), intergenic regions, introns, and mature miRNAs. Clusters from separate libraries were considered overlapping if, based on genome coordinates, they shared >50% of their nucleotides. B. miRNA-interaction 3′UTR sites specific to EBV-infected LCLs. miRNA-interaction sites present in at least two of the five LCL PAR-CLIP libraries were compared to miRNA-interaction sites present in two EBV-negative PEL PAR-CLIP libraries. 109 sites in 3′UTRs contain seed matches to only EBV miRNAs and are highly specific to EBV-infected LCLs. C and D. Breakdown of 3′UTR (C) and CDS (D) sites assigned to individual EBV miRNAs.

The 153,007 clusters identified in all five PAR-CLIP libraries represent ∼23,000 unique CLIPed sites that were present in at least two of the five libraries. Clusters varied in length, between 8 nt to ∼200 nt, with >95% of clusters between 18–31 nt in length (Tables S10–S14), and mapped to 3′UTRs, coding regions, 5′UTRs, introns, miRNAs, and intergenic regions. Congruent with previous reports showing that miRNAs predominantly act on 3′UTRs [10], [48], we found that clusters mapping to regions that were identified in multiple PAR-CLIP libraries were biased toward 3′UTRs (Figure 2A). Of the ∼23,000 CLIPed sites present in at least two of the five libraries, 32.6% mapped to cellular 3′UTRs. The percent of CLIPed sites mapping to 3′UTRs increased to 35.0% and 44.3% when we examined CLIPed sites present in at least three and four libraries, respectively. Of the 2,062 CLIPed sites that were common to all five of the libraries, 53.8% mapped to cellular 3′UTRs compared to 14.3% that mapped to coding regions (CDS) and 0.7% that mapped to 5′UTRs (Figure 2A). A limited number of valid miRNA binding sites for viral miRNAs have previously been identified in coding regions and 5′UTRs [51], [52]; therefore, a subset of clusters mapping to these regions may indeed represent functional miRNA:mRNA interactions.

Prior computational studies predicted that miRNAs not only preferentially target 3′UTRs, but also tend to bind sites located near the termini of 3′UTRs [53], [54]; therefore, we plotted the number of observed clusters against their relative position in a 3′UTR. In agreement with these prior predictions, clusters were generally distributed towards the start and end of 3′UTRs, demonstrating a preference for miRNA binding site positioning (Figure S1B and data not shown).

### EBV-specific miRNA target sites

Based on seed sequence complementarity, 68.6% of all clusters (including 3′UTRs, 5′UTRs, CDS, and intergenic regions) could be assigned to a miRNA that was present in a matched deep sequencing library (Table S1; Tables S10–S14). A total of 7,827 3′UTR miRNA-interaction sites were present in at least two libraries, representing 3,492 different 3′UTRs (Table S6). As an additional filter, we required that the miRNAs assigned to these sites to be represented in at least two of the five small RNA libraries (Tables S2–S5; data not shown). 6,029 (77.0%) of the 7,827 3′UTR sites could be assigned to a miRNA. We identified an additional 2,972 CDS miRNA-interaction sites present in at least two libraries, 89.7% of which could be assigned to a miRNA (Table S7).

To place more stringency on our data and isolate a set of miRNA targets specific to EBV-infected LCLs, we compared the five LCL PAR-CLIP libraries to four PAR-CLIP libraries generated from two B cell primary effusion lymphomas (PEL) that are EBV-negative [55]. 47.3% of the 3′UTR miRNA-interaction sites in LCLs overlapped with clusters generated from the EBV-negative PEL PAR-CLIP libraries (Figure 2B). This amount of overlap was expected since the cellular miRNAs expressed in LCLs and PELs are similar [40], [44], [55]. 52.7% (4,128) of the 3′UTR sites identified in LCLs were specific to EBV-infected LCLs (Table S6, Figure 2B).

**Figure 3:**
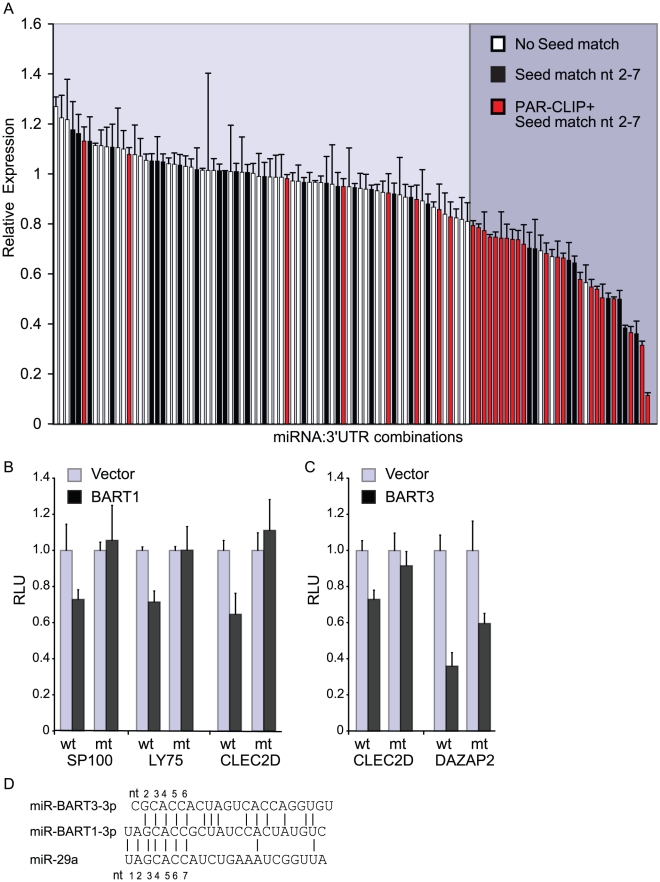
Luciferase reporter assays confirm miRNA-targeted 3′UTRs identified by PAR-CLIP. A. 3′UTRs for 13 genes were inserted into the 3′UTR of firefly luciferase and tested against 11 different miRNA expression vectors for a total of 106 miRNA:3′UTR pairs. 3′UTR reporter plasmids were co-transfected into 293T cells with either EBV or cellular miRNA expression plasmids; lysates were assayed for luciferase activity. All values are relative to an internal renilla luciferase control and then, normalized to luciferase GL3 vector control (no 3′UTR) shown in Figure S3. Reported are the averages of two to five independent experiments performed in triplicates. PAR-CLIP-identified miRNA:mRNA interactions are highlighted in red. 3′UTRs containing a minimal 6mer seed match (nt 2-7) to the assayed miRNA but not identified by PAR-CLIP are shown in black. 32 miRNA:mRNA pairs resulted in >20% luciferase knockdown and are highlighted on the right. B and C. PAR-CLIP identified seed match sites in select 3′UTRs for BART miRNAs were mutated to *NheI* restriction enzyme sites in the luciferase reporter vectors to disrupt miRNA binding. Both the LY75 and DAZAP2 3′UTRs contain two PAR-CLIP-identified sites for miR-BART1-5p and miR-BART3-3p, respectively, and only the site with the highest read count was mutated. Luciferase assays were performed as in (A) and values are shown relative to an internal renilla luciferase control. D. Alignment of miR-BART3-3p, miR-BART1-3p, and cellular miR-29a, which share sequence homology.

We next examined EBV miRNA-targeted sites. 531 (16.2%) of the 7,827 3′UTR miRNA-interaction sites contained seed matches to EBV-B95-8 miRNAs. These 531 sites were present in 494 different 3′UTRs (Table S6). Furthermore, of the miRNA-interaction sites assigned to EBV miRNAs, 70.2% (373 out of 531 sites) were specific to EBV-infected LCLs and therefore, represent high-confidence target sites. A subset of the remaining EBV miRNA-interaction sites that overlapped with the PEL PAR-CLIP libraries can be explained by the fact that EBV miR-BART1-3p shares seed homology (nt 2-7) to cellular miR-29a/b, which is expressed in both LCLs and PEL (Figure 3D; Table S2-S5) [40], [44], [55]. Consequently, a number of miRNA-interaction sites assigned to miR-BART1-3p are also assigned to miR-29a/b (Tables S6, S7, S10–S14). In fact, a large proportion of the miRNA-interaction sites assigned to EBV miRNAs could also be assigned to cellular miRNAs; however, the sites that were assigned to both viral and cellular miRNAs were, on average, longer than the sites assigned only to viral miRNAs (Table S6), and therefore, could potentially represent adjacent miRNA binding sites within a RISC-accessible region on a target mRNA. We identified 129 miRNA-interaction sites that contained canonical seed matches for only EBV miRNAs, and could not be assigned to any cellular miRNAs. Of these 129 sites, 109 (84.5%) were specific to EBV LCLs, and thus represent a highly stringent set of EBV-specific, EBV miRNA targets (Figure 2B; Table S6).

Figure 2C shows the breakdown of the 531 3′UTR sites assigned to individual EBV miRNAs. We identified putative 3′UTR target sites for all highly expressed EBV miRNAs (Figure 2C). We also identified sites for miR-BART2-3p, miR-BART3-5p, and miR-BART4-3p, which are all annotated as miRNA passenger strands (miRNA*) (Tables S2–S5); however, these miRNA*s showed significantly fewer seed matches to PAR-CLIP clusters than the mature EBV miRNAs (Figure 2C). Individual EBV miRNAs had on average ∼40 high-confidence target sites in 3′UTRs, ranging from 14 target sites for miR-BHRF1-3 to 88 target sites for miR-BART2-5p (Figure 2C). We also observed clusters in coding regions with seed matches to viral miRNAs, although compared to 3′UTR sites, the overall number of assigned CDS sites was substantially lower (Figure 2D). In contrast to the previously reported 44 candidate EBV miRNA targets [35], which represent only ∼1% of the potential EBV miRNA targetome, the number of PAR-CLIP identified EBV miRNA seed match sites is much closer to bioinformatic predictions [39] arguing that individual miRNAs can target more than 100 different mRNAs. Thus, including 3′UTR sites, CDS sites, and sites with seed matches to EBV miRNA*s, which are all functional in certain settings [56], [57], the PAR-CLIP-identified EBV-B95-8-miRNA targetome consists of a total of 630 different cellular mRNAs.

The majority of miRNA-interaction sites mapped to cellular, not viral, transcripts, and contained potential seed match sites for either viral or cellular miRNAs (Tables S10–S14). In fact, <0.2% of sites mapped to the EBV genome (discussed below), none of which contained seed match sites for any of the EBV miRNAs. Thus, EBV-B95-8 miRNAs appear to exclusively target cellular transcripts during latent infection, in support of a scenario in which EBV miRNAs facilitate reprogramming of the cellular environment to establish and/or maintain latency.

### PAR-CLIP identifies previously reported miRNA targets

To validate PAR-CLIP-identified targets, we initially queried our data for previously published miRNA targets. First, we examined the Ago2-based RIP-ChIP dataset from Dölken et. al. [35]. These investigators reported 2,337 genes that were enriched in Ago2-immunoprecipitations from six different human B cell lines [35]. Since all of these genes may not be expressed in LCLs, we therefore focused on genes with detectable clusters present in at least two PAR-CLIP libraries. 1,252 genes of the 2,337 genes were represented in the LCL PAR-CLIP libraries. Of these 1,252 represented genes, 91.9% had high-confidence PAR-CLIP-identified miRNA seed match sites (minimum 7mer1A) in their 3′UTRs or CDSs (Tables S6, S7). These results indicate that PAR-CLIP indeed captures a large proportion of previously reported B cell miRNA:mRNA interactions (Figure S2A–C).

We next focused on a single cellular miRNA, miR-155, which is implicated in a number of B cell malignancies. miR-155 is among the most highly expressed cellular miRNAs in LCLs (Figure 1) and is essential for the growth of LCLs *in vitro* [20]. Additionally, miR-155 targets are important to oncogenic herpesvirus biology as both KSHV and MDV encode functional miR-155 analogs [21], [22], [23], [24]. In terms of cellular mRNA targets, miR-155 has been extensively characterized by a number of different approaches, including microarray and proteomics approaches, either through over-expression or knockdown [21], [22], [58]–[61]. Although these prior studies were performed on a variety of different cell types (i.e. epithelial versus B cells) and backgrounds (i.e. mice versus human), and therefore represent a diverse set of miR-155 targets that may not be specific to LCLs, we nevertheless compiled a list of reported miR-155 targets (493 total candidate and validated targets) and compared this list to the high-confidence miR-155-assigned miRNA interaction sites (363 sites representing 349 3′UTRs) presented in Table S6. 84 (24.1%) of the miR-155-assigned 3′UTRs identified by PAR-CLIP were previously reported as potential miR-155 targets (Figure S2D and Table S8). This number rose to 93 when we included high-confidence miR-155-assigned CDS sites from Table S7. We identified 23 of the top 100 miR-155-affected genes determined by pSILAC [58] and 45 of the 149 miR-155 target 3′UTRs examined by Xu et. al. using luciferase reporter assays [60] (Table S15). We also compared our PAR-CLIP miR-155-assigned targets to *in silico* target predictions. TargetScan (v5.1) predicted that 241 (69%) of the 349 miR-155-assigned 3′UTRs were targets of miR-155 [11], [39], [53] (Table S8 and data not shown).

Next, we investigated previously reported targets of EBV miRNAs. To date, 52 candidate targets of EBV miRNAs have been proposed, ten of which have been further validated by luciferase reporters or functional assays [4], [27]–[31], [35]–[38]. We identified EBV miRNA binding sites (minimum nt 2-7 seed match) in the 3′UTRs of 14 of the 44 candidate EBV target genes reported by Dölken et. al.; however, only three of these miRNA:mRNA interactions met our stringent criteria outlined above of being present in at least two libraries with a minimal seed match of 7mer1A (Table S9). The remaining 30 candidate EBV targets may contain seed match sites for the 17 BART miRNAs that are not encoded by EBV B95-8. Five of the ten validated EBV miRNA targets were identified in PAR-CLIP clusters (Tables S10-14), and of these, one target, IPO7, was confirmed as a target of miR-BART3 (Table S9). We also identified the pro-apoptotic tumor suppressor Bim (BCL2L11) as a putative target of miR-BART4 and miR-BART15. Bim was previously reported to be targeted by multiple BART miRNAs [38]. Interestingly, APAF1, a component of the apoptosome, was identified as a strong candidate, EBV-specific target of EBV miR-BART3-3p (Table S6). This further supports a pro-survival, anti-apoptotic role for EBV miRNAs, as previously suggested [37], [38].

MICB and DICER1 were both identified by PAR-CLIP as EBV miRNA targets; however, we identified different miRNAs with seed matches to clusters mapping to these 3′UTRs than what has been previously reported. DICER1 is a reported target of miR-BART6 [36], which is deleted in EBV B95-8. Therefore, we can only say that DICER1 is likely targeted by additional EBV miRNAs, including miR-BART1-5p, miR-BART1-3p, miR-BART3-3p, and miR-BHRF1-1 (Table S9). miR-BART2-5p, which is reported to target MICB [28], is expressed in EBV B95-8 LCLs; however, we did not identify any clusters near the reported miR-BART2-5p binding site in any of the five PAR-CLIP libraries. Instead, we identified seed match sites for miR-BART3 and miR-BART1-3p (Table S9). The reasons for this discrepancy are unclear, but potentially include cell type differences or limits in sequencing depth.

The two remaining cellular genes reportedly targeted by EBV miRNAs, CXCL11 and PUMA, are not detectably expressed in LCLs [29], [37], [62]. However, we did identify a miR-BART1-3p seed match site in the CXCL10 3′UTR (Table S6), which, like CXCL11, is one of three chemokines and T cell attractants that activates the CXCR3 receptor on T cells [29].

### PAR-CLIP identifies functional miRNA targets

To experimentally validate miRNA targets, we cloned 13 PAR-CLIP-identified target 3′UTRs into luciferase vectors and performed dual luciferase reporter assays following ectopic viral or cellular miRNA expression in 293T cells. These 13 diverse 3′UTRs (i) were identified as putative EBV miRNA targets in at least two of the five PAR-CLIP libraries, (ii) contained seed match site(s) for at least one EBV miRNA, and (iii) represent targets of potential interest to EBV biology.

We tested eleven miRNA expression vectors, including six viral miRNAs (miR-BART1, miR-BART2, miR-BART3, miR-BART4, miR-BHRF1-1, and miR-BHRF1-2) and five cellular miRNAs or miRNA clusters (miR-155, miR-146a, miR-128, miR-17/92, and miR-106b/25) against the panel of 3′UTRs. A total of 106 miRNA:3′UTR combinations were tested, 29 of which were identified by PAR-CLIP (Figure 3A, red bars); the other 77 combinations tested served as controls. We observed significant knockdown (>20%) of luciferase expression for 21 out of the 29 (72.4%) PAR-CLIP-identified miRNA:3′UTR pairs, confirming these 3′UTRs as functional miRNA targets. Three additional PAR-CLIP-identified miRNA:3′UTR combinations showed modest but statistically significant knockdown (p<0.05, Figure S3), indicating that ∼83% of assigned miRNA:3′UTR interactions are functional. Five of the 29 PAR-CLIP-identified miRNA:3′UTR pairs showed no knockdown. Thus, using our approach, we can correctly identify a functional miRNA-binding site and the targeting miRNA in ∼75% of cases.

In total, 32 miRNA:3′UTR pairs resulted in >20% luciferase knockdown. Sequence analysis of these 3′UTRs revealed that, in addition to the 21 PAR-CLIP-identified miRNA binding sites, eight seed matches to tested miRNAs were present, which likely explain the observed knockdown (nt 2-7 seed match) (Figure 3A, black bars). However, the presence of a seed match alone was not enough to result in luciferase knockdown. In fact, 59 of the 106 assayed miRNA:3′UTR combinations harbored seed matches, and 27 (46%) of these 59 combinations did not result in any knockdown. Furthermore, when we ranked the miRNA:3′UTR combinations by increasing level of knockdown from left to right (Figure 3A), we find that PAR-CLIP-identified combinations (red bars) cluster together towards the right, while miRNA:3′UTR combinations harboring only seed matches (black bars) are more randomly distributed. These results clearly demonstrate that PAR-CLIP effectively captures functional miRNA target sites.

Three miRNA:3′UTR combinations that were not assigned by PAR-CLIP and lacked miRNA seed matches also resulted in significant luciferase knockdown (Figures 3 and S3). To determine whether non-canonical miRNA targeting might explain the knockdown, we investigated clusters mapping to these three 3′UTRs for potential miRNA target sites using RNAhybrid to calculate binding energy and extent of pairing [63]. We identified two miRNA:3′UTR pairs that exhibited single G:U base pairs in the seed region (miR-BART3-3p/PELI1; miR-BART3-3p/PDE7A). Furthermore, both of these sites with predicted G:U pairs occurred at locations that coincided with PAR-CLIP clusters not assigned to any miRNAs, indicating that these are likely genuine EBV miRNA target sites (data not shown).

Of the 12 PAR-CLIP-identified target 3′UTRs tested against EBV miRNAs, 11 3′UTRs (BACH1, CLEC2D, CLIP1, DAZAP2, KDM4B, LY75, OTUD1, PDE7A, PELI1, SP100, ZNF451) resulted in luciferase inhibition following ectopic viral miRNA expression (Figure S3). These 11 experimentally validated EBV miRNA target genes more than double the current list of reported EBV miRNA targets, making this the largest set of validated EBV miRNA targets to date. Five of the 3′UTRs, including BACH1, CLEC2D, LY75, OTUD1, and PDE7A, were targeted by more than one EBV miRNA. Among these, BACH1, a published target of miR-155 and KSHV miR-K11 [21], [22], can also be targeted by multiple EBV miRNAs (Figure S3). Interestingly, we identified seed matches for both the 3p and 5p strands of miR-BHRF1-2 in clusters mapping to the BACH1 3′UTR, although these sites were identified in only the EF3D-AGO2 PAR-CLIP library (Table S10). Mutational studies on the BACH1 3′UTR indicate that some of these identified binding sites for miR-BHRF1-2 are in fact functional (J.G. Powers and B.R. Cullen, unpublished observations).

To demonstrate that the knockdown of luciferase expression is indeed due to a miRNA:target site interaction, we mutated the seed match sites for BART1 and BART3 miRNAs in four cellular 3′UTRs (SP100, LY75, CLEC2D, DAZAP2). LY75 and DAZAP2 have two PAR-CLIP-identified sites assigned to miR-BART1-5p and miR-BART3-3p, respectively (Table S6); therefore, we mutated the sites with the greatest read counts (Tables S10–S14). With the exception of DAZAP2, mutation of the individual seed match sites fully abrogated the miRNA effect on the reporters (Figure 3B–C). Mutation of the major miR-BART3-3p-assigned site in DAZAP2 only partially relieved the inhibitory effect of BART3 on this 3′UTR (Figure 3C), suggesting that the second PAR-CLIP-identified site for miR-BART3-3p (Table S6) may further contribute to the targeting of this 3′UTR. Interestingly, the CLEC2D 3′UTR contains a short stretch of nucleotides that can be occupied by either miR-BART1-3p or miR-BART3-3p and mutation of this single site abrogates miRNA activity for both of these EBV miRNAs (Figure 3B–C). This result is explained by the partial seed homology shared by miR-BART1-3p and miR-BART3-3p (Figure 3D). In contrast, the DAZAP2 3′UTR, which is targeted by miR-BART3-3p, is not targeted by this miRNA (Figure S3). These results demonstrate that miR-BART1-3p and miR-BART3-3p can have overlapping as well as distinct target mRNAs.

### BHRF1 miRNA deletion mutants reveal high confidence targets

Deletion of EBV BART miRNAs reportedly has little to no effect on the outgrowth of LCLs *in vitro*; however, deletion of all three BHRF1 miRNAs results in significant inhibition of LCL outgrowth and proliferation and also affects EBV latent gene expression [32]–[34]. Arguably, then, BHRF1 miRNAs are likely to have targets that are involved in B cell transformation and/or the establishment of latent EBV infection. We therefore focused on the viral BHRF1 miRNAs and generated LCLs infected with miRNA-knockout viruses lacking either miR-BHRF1-1 or miR-BHRF1-3. These miRNA-knockout viruses, and the phenotypes associated with mutational inactivation of the individual BHRF1 miRNAs, have been described in detail elsewhere [32].

**Figure 4:**
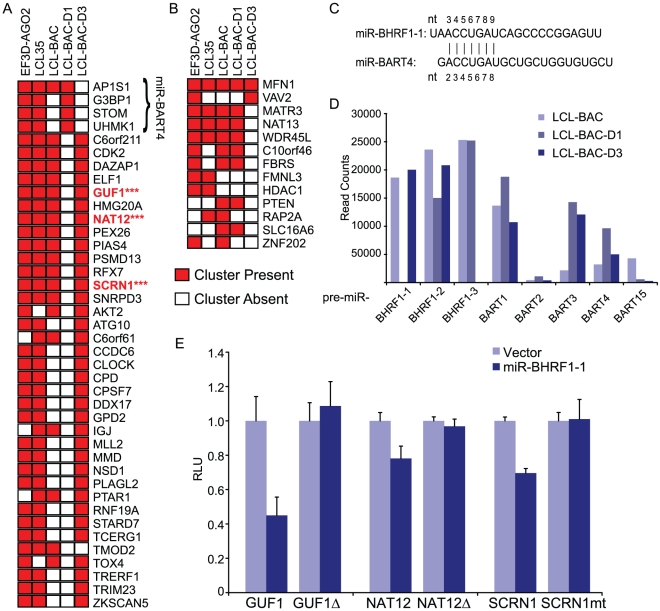
High confidence targets of EBV miR-BHRF1-1 and miR-BHRF1-3. A. 3′UTR clusters assigned to miR-BHRF1-1 (minimum seed match: 7mer1A) that were present in at least three of the five PAR-CLIP libraries and are specific to EBV-infected LCLs are shown in red with annotated gene symbols. Clusters that were absent from the LCL-BAC-D1 PAR-CLIP library are shown in white, and therefore, represent high confidence targets for miR-BHRF1-1. The four miR-BHRF1-1-assigned clusters present in LCL-BAC-D1 can also be assigned to miR-BART4-5p. B. Similar to A, 3′UTR clusters assigned to miR-BHRF1-3 that were present in at least two PAR-CLIP libraries are shown in red; clusters absent from the LCL-BAC-D3 library are shown in white. C. Alignment of miR-BHRF1-1 and miR-BART4-5p, which share an off-set seed sequence. D. EBV pre-miRNAs detected in LCL-BAC, LCL-BAC-D1, and LCL-BAC-D3 as determined by deep sequencing. Reported are the total number of reads mapping to each EBV pre-miRNA. E. PAR-CLIP identified seed match sites to miR-BHRF1-1 in three 3′UTRs (***highlighted in A) were deleted (GUF1 and NAT12) or mutated to an *NheI* restriction enzyme site (SCRN1) in luciferase reporter vectors to disrupt miRNA binding. Luciferase assays were performed as in Figure 3 and values are shown relative to an internal renilla luciferase control.

As described above, we generated PAR-CLIP libraries for LCL-BAC-D1 (lacking miR-BHRF1-1) and LCL-BAC-D3 (lacking miR-BHRF1-3). Small RNA deep sequencing libraries were generated in parallel (data not shown). Deletion of either miR-BHRF1-1 or miR-BHRF1-3 had no major effects on the expression of other viral miRNAs as determined from deep sequencing data (Figure 4D). To identify high confidence targets for these two viral miRNAs, we compared 3′UTR sites that were assigned to each of these miRNAs in the five PAR-CLIP libraries that were specific to EBV-infected LCLs (Table S6). We were stringent in our analysis and required that sites assigned to miR-BHRF1-1 be present in at least three out of the five libraries. 40 3′UTR sites met these criteria (Figure 4A). As expected, 36 of the 3′UTR sites were absent from the LCL-BAC-D1 library, and therefore represent highly stringent, high confidence targets of miR-BHRF1-1 (Figure 4A). For miR-BHRF1-3, only six target 3′UTR sites were identified in three or more PAR-CLIP libraries. When we lowered the cutoff to two or more libraries, a total of 13 3′UTR sites were identified as miR-BHRF1-3 targets, and 11 of these were absent from the LCL-BAC-D3 library. Together, these data indicate that in ∼89% of cases, the correct miRNA is assigned to a PAR-CLIP-identified target site. This number is comparable to the ∼75% of miRNA target sites that were correctly assigned as determined from the 106 miRNA:mRNA combinations tested by luciferase reporter assays (Figure 3).

We selected three 3′UTRs (GUF1, NAT12, and SCRN1) that were identified as high confidence targets for miR-BHRF1-1 for further analysis by luciferase reporter assays. Co-transfection of a miR-BHRF1-1 expression plasmid with each 3′UTR reporter resulted in knockdown of luciferase expression (Figure 4E). This knockdown was specific to the miR-BHRF1-1-assigned target region since mutation of the seed match site (SCRN1) or deletion of the target region (GUF1 and NAT12) abrogated the effect of miR-BHRF1-1 (Figure 4E).

### BHRF1 miRNAs use 5′ canonical seeds for targeting

Surprisingly, miR-BHRF1-3 had only a few assigned target 3′UTRs despite the fact that this miRNA was expressed at levels comparable to miR-BHRF1-1 in LCL-BAC as detected by deep sequencing (Figure 4D). Quantitative analysis of EBV miRNAs in LCLs by qRT-PCR has also shown that the three BHRF1 miRNAs are expressed at similar copy numbers per cell [8]. Even miR-BART2-5p and miR-BART15, which were ∼50 times less abundant than miR-BHRF1-3 according to read counts, had over four times as many assigned target 3′UTRs (Figures 2C and 4D). We investigated whether miR-BHRF1-3 might target regions other than 3′UTRs, such as 5′UTRs or coding regions (CDS); however, there were no major differences in the distribution of clusters for LCL-BAC-D3 compared to the other four libraries (data not shown), and there was no preference in miR-BHRF1-3 seed match sites in CDS compared to 3′UTRs (Figure 2).

**Figure 5:**
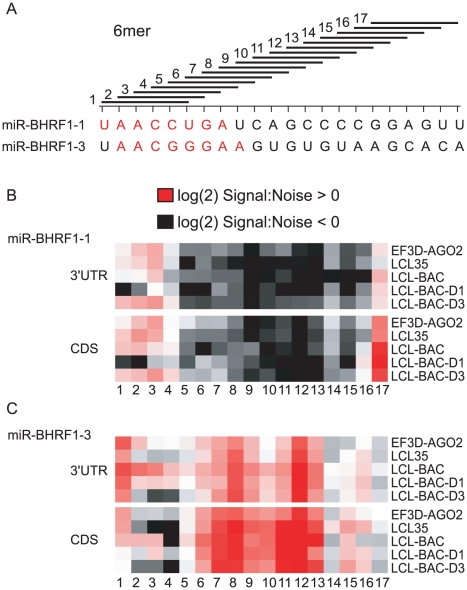
BHRF1 miRNAs target cellular mRNAs through seed match pairing. Consecutive 6mer sequences were examined for pairing with clusters mapping to 3′UTRs and coding regions. A. Schematic showing the interrogated 6mers. The sequences for miR-BHRF1-1 and miR-BHRF1-3 are shown below; highlighted in red are the regions of each miRNA that show enrichment over background in pairing with clusters (nt 1-8 for miR-BHRF1-1 and nt 2-9 for miR-BHRF1-3). B. and C. 6mers from miR-BHRF1-1 and miR-BHRF1-3, respectively, were analyzed for matches to clusters in 3′UTRs or CDSs. Enrichment for a match is indicated in red, while absence of a match is indicated in grey. Numbers below indicate the 6mer sequence described in (A).

We therefore examined PAR-CLIP clusters for miRNA binding sites outside of the canonical 5′ seed match site. We analyzed strings of 6mers present in miR-BHRF1-1 and miR-BHRF1-3 sequences for perfect matches to PAR-CLIP-identified clusters. Notably, we observed enrichment in matches to 6mers encompassing nt 1-6, 2-7, and 3-8 in all four LCLs expressing miR-BHRF1-1 (Figures 1E, 4D, and 5B). As expected, there was a lack of 6mer 5′ seed matches to miR-BHRF1-1 specifically in the LCL-BAC-D1 library, providing strong evidence that miR-BHRF1-1 predominantly uses a canonical 5′ seed of nt 2–7 or nt 2–8, or an off-set 5′ seed of nt 3-8, for mRNA targeting (Figure 5). We extended our analysis to coding regions, which contain documented miRNA target sites [50], [51], [56]. A similar pattern of 5′ seed match enrichment was observed in LCLs expressing miR-BHRF1-1; 21 clusters mapping to coding regions contained 5′ canonical seed matches to miR-BHRF1-1 and were absent from the LCL-BAC-D1 library. Thus, miR-BHRF1-1 binds to CDS sites as well as 3′UTRs.

A slightly different pattern of 6mer matches was observed for miR-BHRF1-3. 6mer matches were present in regions outside of the canonical 5′ seed match region, particularly between nt 6-13; however, a similar number of matches to these regions were also detected in clusters in the LCL-BAC-D3 library. Only the 5′ seed region showed the expected pattern: present in LCLs that express miR-BHRF1-3 (Figures 1 and 4D) and absent from LCL-BAC-D3 (Figure 5C). We did not observe this pattern for coding regions, suggesting that miR-BHRF1-3 targets are largely restricted to 3′UTRs. Since miR-BHRF1-3 contains three G's within the seed region (Figure 5A), we also investigated whether this miRNA might use G:U pairing or contain a bulge in the seed region, but did not observe any conditions which supported these types of pairing (data not shown). Although we cannot rule out other types of atypical miRNA pairing, these data indicate that miR-BHRF1-3 predominantly uses a canonical 5′ seed (minimally nt 2-8) to target 3′UTRs. It is possible that the low target numbers observed might be due to an unfavorable stoichiometric ratio between miR-BHRF1-3 and a small number of high-affinity target mRNAs.

### Redundancy in EBV miRNA targeting

Interestingly, all four miR-BHRF1-1-assigned clusters present in the LCL-BAC-D1 library (AP1S1, G3BP1, STOM, and UHMK1) could also be assigned to miR-BART4 (Table S6 and data not shown). In fact, of the 40 3′UTRs shown in Figure 4A, 35 could be also be assigned to miR-BART4 with a 6mer (nt 2-7) seed match; however, in all instances, miR-BHRF1-1 exhibited more extensive base pairing (data not shown). Analysis of these two miRNAs revealed sequence identity between nt 3-9 of miR-BHRF1-1 and nt 2-8 of miR-BART4 (Figure 4C). As miR-BART4 expression in LCL-BAC-D1 was not significantly affected by deletion of miR-BHRF1-1 (Figure 4D), and 36 clusters are absent in LCL-BAC-D1, these 3′UTRs likely represent bona fide miR-BHRF1-1 targets; however, this does not exclude these 3′UTRs from also being targeted by miR-BART4 at a low level. Conversely, additional miR-BART4-assigned targets present in LCL-BAC-D1 could also be bound by miR-BHRF1-1 to some extent.

These observations prompted us to investigate whether additional viral miRNAs might share common targets. We found that miR-BART1-3p, which shares 100% seed sequence homology with cellular miR-29a/b/c [46], and miR-BART3-3p exhibit extensive sequence homology, sharing 14 out of 22 nucleotides (Figure 3D). Similar to miR-BHRF1-1 and miR-BART4, miR-BART1-3p and miR-BART3-3p share an off-set seed and consequently, a portion of the targets for miR-BART3-3p can be assigned to both these miRNAs as well as cellular miR-29a/b/c with a minimum seed match of nt 2-7 (data not shown). At least some of these sites can be occupied by either miR-BART1-3p or miR-BART3-3p. For example, we confirmed CLEC2D (LLT1), a ligand for natural killer (NK) cells [64], as a target for both of these viral miRNAs using luciferase reporter assays (Figure 3D).

### Cellular pathways targeted by EBV miRNAs

To identify pathways that are potentially regulated by EBV miRNAs, we used PANTHER (Protein Analysis Through Evolutionary Relationships) to examine cellular pathways enriched for virally-targeted genes. Using either the full miRNA targetome identified by PAR-CLIP or the human genome as a reference list, we mapped genes that were assigned as putative targets of EBV miRNAs (3′UTR and/or CDS) in at least two of the five PAR-CLIP libraries (Tables S6, S7). The top 20 cellular pathways potentially targeted by EBV miRNAs are listed in Table 2. These included p53 feedback pathways, B cell signaling, oxidative stress response, and apoptosis, all of which are clearly relevant to EBV infection. Based on this analysis, and further supported by the diversity of high confidence targets that we identified for individual BHRF1 miRNAs (Figure 5), it is evident that EBV miRNAs have not evolved to target one specific cellular pathway and instead, likely have multiple functions.

**Table 2:** Pathways targeted by EBV-B95-8 miRNAs.

	# Ref. Genes		EBV miRNA-targeted 3′UTRs			EBV miRNA-targeted 3′UTRs and CDS		
Pathways	HG	PC	# genes	P value vs HG	P value vs PC	# genes	P value vs HG	P value vs PC
p53 pathway feedback loops 2	52	51	9	7.40E-06	5.23E-04	12	1.49E-07	4.42E-05
T cell activation	102	88	11	5.76E-05	2.14E-03	13	2.82E-05	1.77E-03
B cell activation	82	65	9	2.35E-04	2.72E-03	9	1.32E-03	1.25E-02
p53 pathway	113	104	11	1.40E-04	7.28E-03	13	7.84E-05	6.99E-03
Interleukin signaling pathway	161	104	10	7.19E-03	1.86E-02	10	3.26E-02	7.31E-02
PDGF signaling pathway	159	139	12	6.89E-04	2.23E-02	13	1.84E-03	5.43E-02
Integrin signaling pathway	181	139	12	2.03E-03	2.23E-02	13	5.40E-03	5.43E-02
p53 pathway by glucose deprivation	25	24	4	3.58E-03	2.26E-02	4	8.36E-03	4.81E-02
Parkinson disease	100	88	8	3.68E-03	4.35E-02	9	4.84E-03	6.41E-02
Coenzyme A biosynthesis	6	8	2	9.79E-03	4.92E-02	2	1.55E-02	7.54E-02
Cell cycle	22	19	3	1.75E-02	5.28E-02	3	3.27E-02	9.32E-02
Inflammation mediated by chemokine and cytokine signaling pathway	283	203	14	1.14E-02	6.93E-02	16	1.86E-02	1.15E-01
PI3 kinase pathway	115	81	7	2.48E-02	7.00E-02	9	1.14E-02	4.23E-02
Ubiquitin proteasome pathway	70	67	6	8.21E-03	7.86E-02	6	2.42E-02	1.79E-01
General transcription by RNA polymerase I	20	11	2	8.72E-02	8.55E-02	2	1.30E-01	1.28E-01
Interferon-gamma signaling pathway	29	24	3	3.53E-02	9.10E-02	3	6.39E-02	1.54E-01
Insulin/IGF pathway-protein kinase B signaling cascade	35	59	5	5.59E-02	1.22E-01	7	9.84E-02	5.17E-02
Apoptosis signaling pathway	123	112	8	1.20E-02	1.26E-01	8	4.21E-02	2.98E-01
Ras Pathway	79	77	6	1.42E-02	1.28E-01	6	3.98E-02	2.68E-01
Hypoxia response via HIF activation	32	29	3	4.51E-02	1.38E-01	4	1.90E-02	8.29E-02

HG  =  Homo sapien genes (19,911 total mapped genes).

PC  =  PAR-CLIP genes detected in one or more libraries (11,057 total mapped genes).

### The miR-17/92 cluster targets viral transcripts

**Figure 6:**
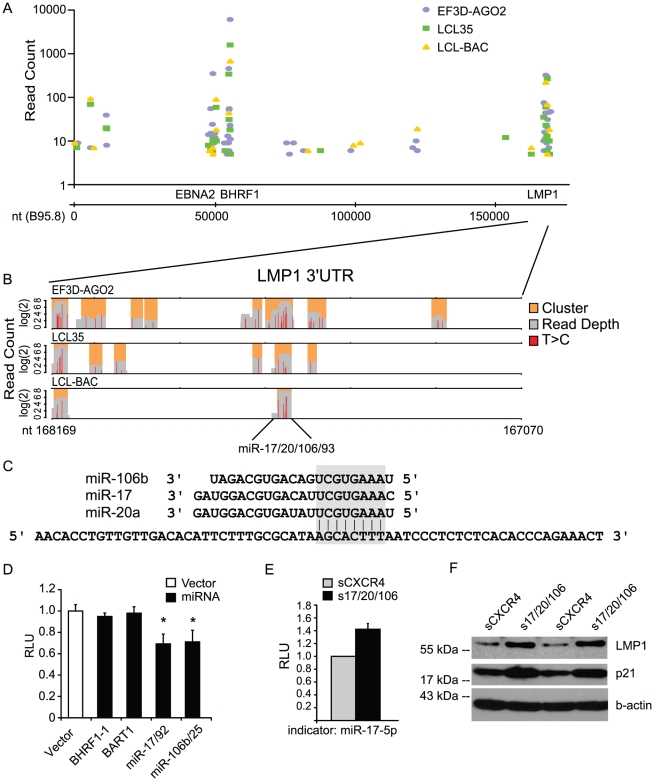
LMP1 is targeted by the miR-17/92 cluster. A. EBNA2, BHRF1, and LMP1 transcripts are targeted by miRNAs in LCLs. Shown are clusters mapping to the EBV B95-8 genome in three PAR-CLIP libraries. Clusters mapping to non-coding RNAs are not shown. B. Multiple Ago2-interaction sites are present in the LMP1 3′UTR. Shown are read groups (grey), clusters (orange), and T>C conversions (red) present in reads mapping to the LMP1 3′UTR in three PAR-CLIP libraries. C. The LMP1 3′UTR contains an extensive seed match to the miR-17/20/106/93 family. Shown are the mature miRNA sequences for miR-106a, miR-17, and miR-20a as identified by deep sequencing and the sequence of the LMP1 3′UTR cluster containing the seed match site. This site was also identified in LCL-BAC-D1 and LCL-BAC-D3 PAR-CLIP libraries (Tables S13 and S14). D. miR-17/92 and miR-106b/25 inhibit the LMP1 3′UTR reporter. The LMP1 3′UTR luciferase reporter and renilla luciferase internal control vector were co-transfected into 293T cells with indicated miRNA expression plasmids. *By Student′s t test, p<0.01 compared to vector control. E. Inhibition of endogenous miR-17/20/106 using a sponge. LCL35 was transduced with pLCE-CXCR4s (control sponge, CXCR4s) or pLCE-miR-17/20/106s (sponge for miR-17/20/106) and FAC-sorted for high GFP expression. To assay miR-17/20/106 activity, cells were transduced with a firefly luciferase indicator to miR-17-5p and renilla luciferase as an internal control. For luciferase assays in D and E, lysates were harvested 48-72 hrs post-transfection or post-transduction and assayed for luciferase activity using the dual luciferase assay kit (Promega). All values are reported as the average of at least two experiments performed in triplicate with standard deviations and are relative to renilla luciferase (RLU  =  relative light units). F. Inhibition of endogenous miR-17/20/106 in LCL35 increases the protein levels of LMP1 and p21 (CDKN1A), a known target of miR-17/20/106. LCL35 was transduced with pLCE-CXCR4s or pLCE-miR-17/20/106s, FAC-sorted for high GFP expression, and lysates harvested 10 days post-FACS for Western blot analysis. Lanes were loaded in duplicate. Beta-actin is shown as a loading control.

Previous studies have reported that the latent viral transcripts, LMP1 and LMP2A, are targeted by BART miRNAs [30], [31]. To determine whether additional viral transcripts might be regulated by miRNAs, we aligned PAR-CLIP reads to the EBV B95-8 genome. Almost all of the reads mapped to latent transcripts, including non-coding RNAs such as EBV pre-miRNAs and EBERs (not shown), consistent with the pattern of latent viral gene expression in LCLs (Figure 6A). In fact, we did not identify reads mapping to lytic transcripts, such as BALF5, a published target of miR-BART2-5p [4], [27]. Three major miRNA-targeted regions were identified, including the EBNA2 mRNA, the 3′UTR of BHRF1, and the 3′UTR of LMP1 (Figure 6A).

A total of seven potential miRNA-interaction sites were identified in the LMP1 3′UTR in at least two or more libraries (Tables S10-S14). One of these sites contained a seed match for the cellular miR-17/20/106/93 seed family and was detected in all five PAR-CLIP libraries (Figure 6B-C, Tables S10-S14). miR-17 and miR-20a are two of six miRNAs expressed from the miR-17/92 cluster, while miR-106b and miR-93 are expressed from the miR-106b/25 cluster [65], [66]. Both miRNA clusters are transcriptional targets of c-myc and are expressed in LCLs at moderate to high levels (Tables S2-S5, Figure 1).

To investigate the interaction between the miR-17/20/106/93 seed family and the LMP1 3′UTR, we generated a luciferase reporter containing the full EBV LMP1 3′UTR (pLSG-LMP1). Over-expression of the miR-17/92 cluster or the miR-106b/25 cluster in 293T cells significantly decreased luciferase expression from pLSG-LMP1, indicating these miRNAs are indeed targeting the LMP1 3′UTR (Figure 6D). To further confirm the miR-17/20/106/93 interaction with the LMP1 3′UTR, we introduced a control sponge (sCXCR4) or a miR-17/20/106 sponge (s17/20/106) into LCL35 to disrupt endogenous miRNA activity (Figures 6E and S2). The miR-17/20/106 sponge contains nine imperfect binding sites within the 3′UTR of GFP for miR-17, miR-20a, or miR-106a (Table S15); miR-106b and miR-93 differ from these three miRNAs in their 3′ non-seed sequences (Figure 6C, Table S1-S4). Following transduction, cells were sorted for high GFP expression, indicative of miRNA inhibition [20], and then, cultured for one week. Western blot analysis of LMP1 protein levels showed upregulation of LMP1 when the activity of endogenous miR-17/20/106 family members was inhibited (Figure 6F). We also observed upregulation of p21 (CDKN1A), a known target of miR-17/20/106 in B cells [66], which we also confirmed as a miR-17/20/106 target by PAR-CLIP (Tables S10-S14). Furthermore, we observed a ∼4-fold increase in the levels of LMP1 mRNA in miR-17/20/106-sponged LCL35 compared to control cells by qRT-PCR; the levels of EBNA2 mRNA were not affected (data not shown). Together, these data show that the miR-17/20/106 family can target the LMP1 3′UTR in LCLs during latent EBV infection.

The LMP1 3′UTR has previously been reported to be targeted by multiple EBV BART miRNAs [30], including miR-BART1-5p which is encoded by B95-8; therefore, we also tested EBV miRNAs against the LMP1 luciferase reporter (Figure S4E). We did not, however, observe (i) any cluster mapping to LMP1 bearing a miR-BART1-5p seed match (Tables S10-S14) or (ii) any inhibition of luciferase activity following ectopic expression of miR-BART1-5p in 293T cells, indicating that miR-BART1-5p does not target the LMP1 3′UTR (Figures 6D and S4E). Interestingly, miR-BART3, which does not bear a seed match to any PAR-CLIP identified cluster, inhibited luciferase activity from the LMP1 3′UTR reporter (Figure S4E). We therefore investigated clusters mapping to the LMP1 3′UTR for potential miR-BART3 binding sites. Indeed, one cluster coincided with a predicted non-canonical target site, which may explain the observed luciferase knockdown (data not shown).

In addition to the confirmed miR-17/20/106 site within the LMP1 3′UTR, we identified a second binding site for the miR-17/20/106 seed family in the 3′UTR of BHRF1 (Figure S4; Tables S10-S14). As the cellular miRNAs for miR-17/92 and miR-106b/25 clusters are evolutionarily conserved in mammals, we wondered whether the target sites in the BHRF1 and LMP1 3′UTRs might also be conserved. Therefore, we analyzed BHRF1 and LMP1 sequences from the related rhesus lymphocryptovirus (rLCV; NM_006146) using RNAhybrid and the corresponding miRNA sequences from *Macaca mulatta* obtained from miRBASE v16.0 [63], [67]. Intriguingly, predicted mml-miR-17/20/106 binding sites in both the rLCV BHRF1 and LMP1 3′UTRs were identified, and similar to the hsa-miR-17/20/106 sites in EBV, exhibited extensive pairing in the seed region (Figure S4A-D). These observations provide evidence in support of an evolutionarily conserved role for miR-17/20/106-dependent regulation of LMP1 and BHRF1 transcripts during viral infection.

## Discussion

In this study, we present the EBV and cellular miRNA targetome in LCLs, which includes ∼500 EBV miRNA target sites in 3′UTRs. Using luciferase assays, we experimentally validated 24 miRNA:3′UTR interactions, and 14 target genes of EBV miRNAs. Furthermore, using BHRF1 miRNA mutant viruses, we identified a high confidence set of targets that are specific to EBV-infected LCLs for miR-BHRF1-1 and miR-BHRF1-3. This study represents the largest set of EBV miRNA targets to date, and demonstrates that EBV miRNAs predominantly target cellular genes during latent infection. Presumably, viral miRNAs reprogram the host environment to favor viral persistence. Pathway analysis predicts that many of the genes targeted by EBV miRNAs have roles in p53 feedback loops, B cell activation, , and apoptosis.

We validated several EBV miRNA targets with potential immunomodulatory roles. SP100 and ZNF451 are both components of promyelocytic leukemia (PML) bodies, which are implicated in interferon-induced antiviral defenses [68], [69]. PDE7A is involved in cytokine production and T cell activation [70]. miR-BART3-3p and miR-BART1-3p both target the 3′UTR of CLEC2D (LLT1), a C-type lectin receptor (CLR) and NK ligand [64]. We also confirmed the CLR LY75 (DEC205) as a target of miR-BART1-5p (Figure 3). Additional CLRs including CLEC2B (AICL), an NKp80 ligand and CLEC7A (Dectin-1) [71] were also identified as potential EBV miRNA targets (Tables S6, S10-S14). Interestingly, the CLEC2D, CLEC7A, and LY75 3′UTRs all contain predicted seed match binding sites for other herpesvirus miRNAs, including those expressed by KSHV and HCMV (data not shown). Furthermore, the NK ligand MICB has previously been reported to be regulated by multiple herpesvirus miRNAs, including EBV miR-BART2-5p [28]. We did not detect a binding site for miR-BART2-5p in the MICB 3′UTR by PAR-CLIP (Tables S10-S14); however, we did identify clusters in the MICB 3′UTR with 6mer seed matches to other BART miRNAs, suggesting that MICB may be regulated at some level by EBV miRNAs. Together, these observations suggest that the downregulation of multiple CLRs and NK ligands, to prevent recognition of virally-infected by the host immune system, is a conserved function of herpesvirus miRNAs.

EBV, KSHV, and HCMV miRNAs do not share seed sequence homology and therefore, are not predicted to occupy the same sites on target mRNAs; however, it is conceivable that herpesviruses have evolved to target different RISC-accessible sites on the same mRNAs—specifically those target mRNAs that are of general importance to herpesvirus biology. In fact, of the genes with seed match sites assigned to EBV miRNAs (Tables S6, S7), a significant proportion contain predicted seed match sites for either KSHV or HCMV miRNAs (data not shown). Furthermore, >50% of the 3′UTR sites specifically assigned to cellular miR-155, which is induced by EBV infection and critical for LCL growth [20], overlapped with sites present in KSHV+ PEL PAR-CLIP libraries (Table S6). KSHV expresses the viral miRNA miR-K11, which has perfect seed homology to miR-155 [21], [22], and thus, these overlapping sites also represent putative targets of KSHV miR-K11.

At least 14 EBV miRNAs, including those not encoded by B95-8, share seed sequence homology with cellular miRNAs [46]. Furthermore, this seed sharing for EBV miRNAs is statistically favored and two times more abundant than what is expected to occur by chance [25], [46]. Theoretically, viral miRNA mimicking of cellular miRNA seed sequences allows the miRNA to gain access to and exploit existing host regulatory pathways, thereby potentially skewing the outcome of miRNA regulation by occupying those binding sites. Arguably, miR-BART1-3p, and to some extent miR-BART3-3p as described above (Figure 3D), regulate the same cellular processes as the miR-29 family members, which are moderately to highly expressed in LCLs (Figure 1).

Interestingly, miR-29 is described as both a tumor suppressor and oncogene [72]. In non-B cells, miR-29 is reported to modulate Wnt signaling [73]. In B cells, miR-29b downregulates TCL1, an oncogene that is responsible for the development of aggressive chronic lymphocytic leukemia (CLL) [72], [74]. In EBV-infected B cells, EBV LMP1 induces the expression of miR-29b, thereby indirectly downregulating TCL1 protein levels [75]. miR-29 also targets the MCL1 3′UTR, confirmed here (Tables S10-S14), thereby increasing cell sensitivity to apoptosis [76]. However, over-expression of miR-29 in transgenic mice can also promote B cell proliferation [74]. Thus, disrupting finely-tuned miR-29-regulated processes by EBV miRNAs that mimic the seed sequence of a host miRNA could have significant consequences.

We observed that EBV miRNAs not only exhibit seed homology to cellular miRNAs but also share seed homology amongst themselves (Figures 3D and 4C). This observation arose while interrogating high confidence targets for miR-BHRF1-1; several cellular 3′UTR clusters can be assigned to both miR-BHRF1-1 and miR-BART4 due to off-set seed homology between these two miRNAs. As not all EBV miRNAs are expressed throughout all stages of latency or in all cell types infected by EBV [3], [5]-[7], this may be one way EBV can ensure that important cellular transcripts are nevertheless downregulated.

Three lines of evidence demonstrate that the LMP1 3′UTR is targeted by the c-myc-regulated miRNA clusters miR-17/92 and miR-106b/25. First, we identified binding sites for the miR-17/20/106 seed family within the LMP1 3′UTR by PAR-CLIP (Figure 6). Second, luciferase reporter assays confirmed the interaction, and finally, inhibition of endogenous miRNA expression using a miRNA sponge lead to an increase in LMP1 protein levels (Figure 6). The presence of a cellular miRNA binding site in a viral transcript is counterintuitive, especially since a virus could readily evolve to avoid miRNA targeting. We observed knockdown of a luciferase reporter following ectopic miRNA expression and enhanced expression of LMP1 when miR-17/20/106 miRNAs were inhibited, consistent with the canonical activity of miRNAs [10]. Thus, this target interaction site does not appear to provide any novel function, as has been demonstrated for miR-122 and its interaction with the 5′ UTR of hepatitis C virus (HCV) [77]. We identified a second miR-17/20/106 seed match site in the BHRF1 3′UTR. Furthermore, these miR-17/20/106 seed match sites appear to be highly conserved in the rLCV LMP1 and BHRF1 3′UTRs (Figure S4). EBV and rLCV diverged over 13 million years [3], signifying the importance of these miRNA-interaction sites. The functional relevance of the miR-17/20/106 site within the LMP1 3′UTR is currently unclear. Preliminary studies with viruses in which the miR-17/20/106 binding site is mutated indicate that this site is not essential for B cell transformation *in vitro* (Feederle et. al., unpublished observations). Possibly, the downregulation of LMP1, and also BHRF1, expression by miR-17/20/106 is important during the transition from latency III to latency I *in vivo*. Interestingly, both miR-17/92 and miR-106b/25 are transcriptional targets of c-myc [65], [66]. In latency III LCLs, LMP1-induced NFkB activity is the major force driving cell survival and proliferation. However, in EBV+ BL cells, where LMP1 is no longer expressed, c-myc is the main driver for cellular proliferation [78]–[80]. Thus, binding of c-myc-regulated miRNAs to the LMP1 3′UTR may be one way to transition between these seemingly incompatible growth programs.

In conclusion, our data provides an extensive list of viral and cellular miRNA targets in B cells. Presumably, many of these miRNA:mRNA interactions will be of importance to viral pathogenesis and lymphomagenesis *in vivo* and for the maintenance of viral latency and/or cell survival in culture. The identification of a large set of targets for virally encoded or EBV-induced cellular miRNAs, combined with emerging data demonstrating the deleterious phenotypic effects that arise upon loss of these miRNAs in culture [32]–[34], now sets the scene for efforts to rescue these phenotypes. For example, short interfering RNAs (siRNAs) can be used to inhibit the expression of identified, target mRNAs, thereby elucidating the importance of specific miRNA targets in the establishment and/or maintenance of EBV latency.

## Materials and Methods

### Cell culture and infections

Established lymphoblastoid cell lines (LCLs) are infected with EBV B95-8 or an EBV B95-8 Bacmid [32], [33] (EF3D, EF3D-AGO2, LCL35, SDLCL, LCL-BAC, LCL-BAC-D1, LCL-BAC-D3) and were maintained in RPMI 1640 medium supplemented with 15% FBS and 10 µg/mL gentamicin (GIBCO). LCLs were analyzed three to five months post-infection. SDLCL has been previously described [20]. EF3D-AGO2 cells stably express a FLAG-tagged Ago2 and were generated by transducing EF3D with pMIGw-FLAG/HA-Ago2-GFP (described below) followed by sorting for GFP expression. LCL-BAC-D1 (mutationally inactivated for miR-BHRF1-1 expression) and LCL-BAC-D3 (mutationally inactivated for miR-BHRF1-3 expression) are infected with EBV miRNA-mutant viruses described in detail elsewhere [32], and were generated from the same donor as LCL-BAC.

miRNA-sponged LCLs were generated by transducing LCL35 with pLCE-sCXCR4 (control sponge, described in [81]) or pLCE-s17/20/106, containing nine imperfect target sites for miR-17, miR-20a, or miR-106a in the 3′UTR of GFP [81], and FAC-sorting for high GFP-expressing cells 48-hrs post-infection. FAC-sorting was done at the Duke Cancer Institute Flow Core Facility. Virus for all transductions was produced in 293T cells as described previously [20], [22].

### Small RNA deep sequencing and preparation of PAR-CLIP libraries

Small RNA deep sequencing libraries were generated as previously described [44]. Briefly, 30 µg of total RNA was size-fractionated on 15% TBE-Urea polyacrylamide gels. Small RNAs corresponding to 18–24 nt in length were sequentially ligated to 3′ and 5′ Illumina adapters which are selective for RNAs that have a 5′ monophosphate such as miRNAs. Ligated RNAs were reverse transcribed using SSII (Invitrogen) and cDNAs were PCR amplified (16–22 cycles) prior to sequencing.

PAR-CLIP libraries were generated as described in [50]. PAR-CLIP libraries from EBV-negative primary effusion lymphoma (PEL) cell lines (BCBL1 and BC3) are described in [55]. Briefly, ∼1×10∧9 cells were cultured in the presence of 100 uM 4-thiouridine (4SU) (Sigma) for 16–18 hrs and then, irradiated at UV 365 nm. Cells were lysed on ice in NP40-lysis buffer and cross-linked Ago2:RNA complexes were immunoprecipitated using either mono-clonal anti-FLAG antibodies (clone M2, Sigma) for EF3D-AGO2 or monoclonal antibodies to endogenous Ago2 (clone 9E8.2, Millipore). Ago2-bound RNAs were digested with RNAse T1, radiolabeled, gel-purified, and sequentially ligated to 3′ and 5′ Illumina adapters for deep sequencing. Ligated RNAs were reverse transcribed using SSIII (Invitrogen) and cDNAs were PCR amplified (22-28 cycles). A pilot PCR was performed for each small RNA or PAR-CLIP library to ensure amplification occurred in the linear range. Sequencing was performed at the Duke University IGSP Sequencing Core Facility using an Illumina GAIIx Sequence Analyzer. Sequencing data was submitted to the NCBI Sequence Read Archive (SRA) (GEO Accession # in progress). Processed files can be also accessed online (http://cullenlab.duhs.duke.edu/publications/sup/).

### Bioinformatics

Sequencing reads were pre-processed using the FAST-X toolkit (http://hannonlab.cshl.edu/fastx_toolkit/) to remove 5′ barcode and 3′ adapter sequences. Reads ≥13 nt in length were aligned concurrently to the human genome (hg19) and EBV B95-8 genome (NCBI Accession No. V01555.2) or EBV-B95-8 Bacmid sequence using Bowtie [82]. Up to three mismatches were allowed, and genomic locations with reads falling into the best stratum (i.e. lowest number of mismatches) were kept for further analysis. The number of reads aligned to the human and EBV genomes for PAR-CLIP libraries is reported in Table S1. For miRNA annotation, a maximum of 25 different alignment locations was allowed in order to preserve reads mapping to miRNA families, and a minimum of five reads aligning to a pre-miRNA location described in miRBASE v16.0 [67] was required, given that at least one read aligned with no mismatches.

Further analysis of PAR-CLIP reads was performed using the PARalyzer pipeline described in detail elsewhere [49]. Briefly, reads that aligned to a unique genomic location, after subtraction of T>C mismatches, and overlapped by at least one nucleotide were grouped together. Read groups were analyzed for T>C conversions and nucleotide strings containing a greater likelihood of converted T>Cs than non-converted Ts were extracted as clusters. Clusters require a read depth of at least five reads, exclude genomic repeat regions, include sequence from all reads meeting the T>C conversion criteria, and represent the Ago2:RNA interaction site. Clusters were interrogated for miRNA 5′ canonical seed match sites (≥7mer1A, i.e. nt 2-7 match with an A across from position one of the mature miRNA [10], unless otherwise noted) using miRNA sequences identified in a matched small RNA sequencing library. Seed matches described as 12mer matches also include those matches greater than 12mers. miRNA-interaction sites (Figure 2B-D, Tables S6 and S7) are subsequently defined as genomic regions, 8 nt or longer, present in clusters overlapping in two or more libraries.

### Plasmids, miRNA expression vectors, and luciferase assays

pMIGw-FLAG/HA-Ago2-GFP was generated by PCR-amplifying FLAG/HA-Ago2 from pIRESneo-FLAG/HA-Ago2 [83] and cloning into pMIGw using *BglII* and *XhoI* sites [84]. To generate miRNA expression vectors, ∼300 nt region encompassing each pre-miRNA was PCR amplified from LCL genomic DNA and cloned into pcDNA3 downstream of the CMV promoter or into pLCE [81], [85] in the GFP 3′UTR using *XhoI* and *XbaI* sites. For miR-17/92 and miR-106b/25, regions encompassing the entire pre-miRNA clusters were cloned. To assay miRNA expression, indicator vectors were generated containing a firefly luciferase cassette (pcDNA3-GL3 or pL-CMV-GL3) and two fully complementary binding sites for the miRNA of interest inserted using *XhoI* and *XbaI* sites into the 3′UTR (Table S15). pL-CMV-GL3 and pL-CMV-Rluc, expressing renilla luciferase, are derivatives of pLSG and pLSR [81]. pcDNA3-GL3 and pcDNA3-Rluc are derivatives of pL-CMV-GL3 and pL-CMV-Rluc. To confirm miRNA expression, each miRNA expression vector was tested against its respective indicator (data not shown) as well as empty vector (control in Figure S3).

Target 3′UTRs identified by PAR-CLIP were PCR amplified from LCL genomic DNA using primers in Table S15 and inserted into the 3′UTR of firefly luciferase in pLSG or pcDNA3-GL3 (*XhoI, XbaI*). Luciferase reporters containing the 3′UTR of BACH1 or CDKN1A are previously described [22], [81]. 293T cells were transfected in 24-well plates with 10 ng firefly luciferase reporter (pLSG with indicated 3′UTR), 5 ng pLSR or pCMV-RLuc expressing renilla luciferase, and 1 µg miRNA expression plasmid using Fugene 6 according to the manufacturer's protocol (Roche). 48–72 hrs post-transfection, cells were lysed in 1X passive lysis buffer (Promega) and luciferase expression was assayed using the dual reporter luciferase assay system (Promega). All luciferase assays are reported as the average of two to five independent experiments performed in triplicates, and p-values were determined by Student's t test.

### Primer extension assays

Primer extensions were performed with 10 µg of total RNA using the AMV PE kit according to the manufacturer's protocol (Promega). Oligonucleotides used for probes are listed (Table S15) and were end-labeled using γ32P-ATP and T4 polynucleotide kinase. Reverse transcription products were separated on 15% TBE-urea polyacrylamide gels and exposed to film.

### Western Blot analysis

Cells were lysed in lysis buffer containing 1% NP40, 50 mM Tris, and 150 mM NaCl. Protein concentrations were determined using the BCA protein assay kit (Thermo Scientific) and 10 µg total protein was loaded per lane on a SDS-polyacrylamide gel. Following electrophoresis, proteins were transferred to nitrocellulose and membranes were probed with primary antibodies to LMP1 (monoclonal, clone S12), p21 (c-19, Santa Cruz), or beta-actin (c4, Santa Cruz). Corresponding horse-radish peroxidase (HRP) conjugated secondary antibodies were to mouse (LMP1 and beta-actin) or rabbit (p21). Blots were developed using LumiLight western blot substrate (Roche), for LMP1 or beta-actin, or SuperSignal West Femto maximum sensitivity substrate for p21 (Thermo Scientific) and exposed to film.

### Pathway analysis

Pathway analysis was performed using PANTHER (www.pantherdb.org). 3′UTRs and CDSs assigned to EBV miRNAs that occurred in at least two of the five PAR-CLIP libraries were input using all CLIPed genes (11,057 mapped genes) as the reference list. The human genome (19,911 genes) was interrogated as a second reference list. Biological processes were sorted according to p-value. Reported are the top 20 pathways targeted by EBV miRNAs.

## Supporting Information

Figure S1 **Analysis of PAR-CLIP clusters.** A. Overlap between the five PAR-CLIP libraries. Shown is the percent of clusters (including 3′UTRs, 5′UTRs, CDS, and intergenic sites) within individual libraries that can be identified in at least one other PAR-CLIP library. Clusters from separate libraries were considered overlapping if they shared at least 50% of their nucleotides (also for C and D). B. Clusters are preferentially distributed towards the termini of 3′UTRs. Plotted is the number of PAR-CLIP identified clusters against their relative position in a 3′UTR for LCL35. Similar results were observed for the other libraries (not shown). C. Clusters with high read counts are more consistently captured in multiple libraries than clusters with low read counts. The average PAR-CLIP read value of clusters for any particular set of overlapping clusters was recorded. This value was then plotted by the number of libraries that had a cluster within the set of overlapping clusters. D. Clusters with seed matches to highly expressed miRNAs are more consistently captured in multiple libraries than clusters with seed matches to weakly expressed miRNAs. The miRNA with the highest expression value assigned to any one of the clusters within the set of overlapping clusters was recorded. This value was then plotted by the number of libraries that had a cluster within the set of overlapping clusters.
(EPS)

Figure S2 **PAR-CLIP identifies previously reported miRNA targets.** A-C. Overlap of the 2,337 mRNAs enriched in Ago2-immunoprecipitations from human B cells (Dölken, et. al., 2010) with high-confidence PAR-CLIP-identified miRNA-interaction sites that were present in at least two of the five libraries (3′UTRs and/or CDSs from Tables S6 and S7). Venn diagrams were generated using the online tool from the MIT Whitehead Institute: http://jura.wi.mit.edu/bioc/tools/D. Overlap of previously reported miR-155 targets with high-confidence PAR-CLIP-identified miRNA-interaction sites that were present in at least two of the five libraries (3′UTRs only from Table S6). Also see Table S8.
(EPS)

Figure S3 **Cellular targets of EBV and cellular miRNAs confirmed by luciferase reporter assays.** 3′UTRs for indicated genes were inserted into the 3′UTR of firefly luciferase. 3′UTR reporter plasmids were co-transfected into 293T cells with indicated miRNA expression plasmids; lysates were harvested 72 hrs post-transfection and assayed for luciferase activity. All values are shown relative to an internal renilla luciferase control. Reported are the averages of two to five independent experiments performed in triplicates. (RLU  =  relative light units) miRNA:3′UTR combinations that were identified by PAR-CLIP and show knockdown by luciferase reporter assays are highlighted in dark red; those miRNA:3′UTR combinations that did not respond as predicted by PAR-CLIP are highlighted in pink. Highlighted in grey are 3′UTRs which respond to ectopic miRNA expression but were not identified as targets by PAR-CLIP. *indicates statistically significant knockdown (p<0.05), but does not meet the >20% knockdown cut-off. **3′UTR contains a canonical seed match (minimally nt 2-7) or seed match with G:U pairing (minimally nt 2-8) to the respective miRNA. Two of these predicted miRNA:3′UTR interaction sites with G:U pairing in the seed coincide with PAR-CLIP clusters that could not be assigned to any miRNAs.
(EPS)

Figure S4 **The miR-17/20/106 binding sites in LMP1 and BHRF1 are conserved in rLCV.** A-D. The EBV and rLCV latent transcripts, LMP1 and BHRF1, contain miR-17/20/106 seed match sites in their 3′UTRs**.** Sequences of the LMP1 (A and B) and BHRF1 (C and D) 3′UTRs from EBV and rLCV were analyzed for miR-17/20/106 seed match sites using RNAhybrid. Shown are the PAR-CLIP identified sites for human miR-17/20/106 in EBV mRNAs and the predicted sites for macaque miR-17/20/106 in rLCV mRNAs. E. The LMP1 3′UTR luciferase reporter responds to EBV miR-BART3. The LMP1 3′UTR reporter was co-transfected into 293T cells with indicated EBV miRNA expression plasmids; lysates were harvested 72 hrs post-transfection and assayed for luciferase activity. All values are shown relative to an internal renilla luciferase control. Reported are the averages of at least two independent experiments performed in triplicates. (RLU  =  relative light units)
(EPS)

Table S1 **Deep sequencing reads aligned to the human and EBV genomes.**
(XLS)

Table S2 **miRNAs detected in EF3D-AGO2.**
(CSV)

Table S3 **miRNAs detected in LCL35.**
(CSV)

Table S4 **miRNAs detected in LCL-BAC.**
(CSV)

Table S5 **miRNAs detected in SDLCL.**
(CSV)

Table S6 **High confidence miRNA-interaction sites in 3′UTRs.**
(XLS)

Table S7 **High confidence miRNA-interaction sites in CDSs.**
(XLS)

Table S8 **Previously reported miR-155 targets detected in PAR-CLIP.**
(XLS)

Table S9 **Previously reported EBV miRNA targets.**
(XLS)

Table S10 **miRNA seed matches in EF3D-AGO2 clusters.**
(CSV)

Table S11 **miRNA seed matches in LCL35 clusters.**
(CSV)

Table S12 **miRNA seed matches in LCL-BAC clusters.**
(CSV)

Table S13 **miRNA seed matches in LCL-BAC-D1 clusters.**
(CSV)

Table S14 **miRNA seed matches in LCL-BAC-D3 clusters.**
(CSV)

Table S15 **Oligonucleotide sequences.**
(XLS)
